# Clinical Problems in the Hospitalized Parkinson's Disease Patient: Systematic Review

**DOI:** 10.1002/mds.23449

**Published:** 2011-01-31

**Authors:** Oliver HH Gerlach, Ania Winogrodzka, Wim EJ Weber

**Affiliations:** Section of Movement Disorders, Department of Neurology, Maastricht University Medical CentreMaastricht, The Netherlands

**Keywords:** Parkinson's disease, hospitalization, emergency room, perioperative

## Abstract

The problems Parkinson's disease (PD) patients encounter when admitted to a hospital, are known to be numerous and serious. These problems have been inventoried through a systematic review of literature on reasons for emergency and hospital admissions in PD patients, problems encountered during hospitalization, and possible solutions for the encountered problems using the Pubmed database. PD patients are hospitalized in frequencies ranging from 7 to 28% per year. PD/parkinsonism patients are approximately one and a half times more frequently and generally 2 to 14 days longer hospitalized than non-PD patients. Acute events occurring during hospitalization were mainly urinary infection, confusion, and pressure ulcers. Medication errors were also frequent adverse events. During and after surgery PD patients had an increased incidence of infections, confusion, falls, and decubitus, and 31% of patients was dissatisfied in the way their PD was managed. There are only two studies on medication continuation during surgery and one analyzing the effect of an early postoperative neurologic consultation, and numerous case reports, and opinionated views and reviews including other substitutes for dopaminergic medication intraoperatively. In conclusion, most studies were retrospective on small numbers of patients. The major clinical problems are injuries, infections, poor control of PD, and complications of PD treatment. There are many (un-researched) proposals for improvement. A substantial number of PD patients' admissions might be prevented. There should be guidelines concerning the hospitalized PD patients, with accent on early neurological consultation and team work between different specialities, and incorporating nonoral dopaminergic replacement therapy when necessary. © 2011 Movement Disorder Society

## Introduction

Parkinson's disease (PD) is the second most common neurodegenerative disorder with a life-time risk of 2 percent in men and 1.3 percent in women.^1^ Although the disorder is generally slowly progressive, it does have a major impact on disability and quality of life of affected patients.^2^,^3^ One of the lesser studied aspects of PD is the spectrum of problems PD patients encounter, once they are admitted to a hospital. In our own and others' experience hospital admissions of PD patients are often problematic, especially so when patients are admitted on non-neurological wards.^4^,^5^ Problem areas are exact timing or lack of drug administration, administration of contra-indicated drugs, complications due to immobilization, and psychiatric disorders triggered by the hospital admission.^6^–^8^ As most non-neurologically educated health care personnel are unfamiliar with PD, protocols would be helpful to improve the care of PD patients in such environments.

We recently surveyed the majority of movement disorder specialist neurologists in the Netherlands and found that no specific guidelines or protocols exist to guide caregivers and PD patients in the hospital environment (manuscript in preparation). Before such guidelines can be formulated, one needs to know the prevalence and spectrum of the problems PD patients may experience during their hospital stay. To this end, we systemically reviewed all the existing literature on the problems encountered by the hospitalized PD patients.

## Methods

We systematically reviewed the literature on reasons for emergency room (ER) and hospital admissions in patients with PD, problems encountered during hospitalization of this patient population, and possible solutions for the encountered problems, using the Pubmed database. Last research date was 17 June 2010.

To identify articles, we included (combinations of) keywords: See Table 1 for search details. Subsequently we analyzed the abstracts for relevant articles: as relevant articles we defined those as pertaining to the following four areas:

1. Analysis of prevalence and reasons for ER visits and subsequent admission.
2. Clinical problems during hospital stay.
3. Peri- and postoperative problems.
4. Suggestions for improvement of care for the hospitalized PD patient.

**TABLE 1:** Pubmed search details

Search	Number of hits
Parkinson* and emergenc*	422
Parkinson* and hospitali*	645
Parkinson* and healthcare	892
Parkinson* and hospital admission*	45
Parkinson* and hospital utilization*	4
Parkinson* and resource use	186
Parkinson* and perioperative	93
Parkinson* and preoperative	451
Parkinson* and intraoperative	453
Parkinson* and anesthes*	373
Parkinson* and surgery and cognit*	456
Parkinson* and surgical problems	260
Parkinson* and surgery and apomorphine	289
Parkinson* and postoperative	1231
Parkinson* and surgery and complication*	2241
Medication interruption or drug manipulations OR discontinuation or dose reduction and levodopa	576
Parkinson* and drug withdrawal	607
Parkinson* and lisuride	268
Parkinson* and medication error	59
Amantadine and intravenous*	106
Rotigotine	247
Parkinson* and fracture	236
Parkinson* and nurse	276
Parkinson* and nurse specialist	26
Parkinson* and orthopaedic	94
Parkinson* and orthopedic*	52

We also searched the reference list of each relevant article for other applicable articles.

In our search, there were no language limitations. We excluded articles concerning brain surgery.

## Results

### Emergency Room and Hospital Admissions

Patients with PD often need emergency treatment. There are four studies analyzing the reasons for ER admission,^4^,^9^–^11^ including one case report^11^ (totaling 327 PD patients, Table 2). A total of 16–45% of PD patients visit an ER once a year.^9^,^10^,^12^,^13^ PD patients visit the ER more frequently than their matched reference group (0.6 vs. 0.4; *P* = 0.05).^14^

**TABLE 2:** Emergency room admissions

Study	Inclusion	Exclusion	Number of patients	Design	Control group	ER admissions (%)	Reasons ER visit
Vargas et al., 2007^9^	PD	Hoehn and Yahr Stage 5	144	Retrospective	No	22% in 1 yr	Primarily side effects of anti-Parkinsonian drugs
		Parkinsonism					
		Severe cognitive dysfunction					
Cosentino et al., 2005^10^	PD	Parkinsonism	130	Retrospective	No	22% in 1 yr	Injuries 61%, mainly fractures 37%
		Admissions related to PD					Abdominal pain 6%
							Pneumonia, dysphagia, dyskinesia, epistaxis, hearing loss, pulp disease, teeth extraction, lumbago, pain in joint: all 3%
Martignoni et al., 2004^4^	PD	Parkinsonism	48	Prospective	No	All selected patients	Cardiovascular 27%
							Trauma with fractures 19%
							Chest or abdominal problems 19%
							Neurological (both related and unrelated to PD) 17%
							Head injury 6%
							Hip prothesis displacement 2%
Factor and Molho, 2000^11^	PD		5	Case report	No	All selected patients	Severe motor off periods, dyskinesia, psychosis, acute confusion, panic disorder, pain

ER, emergency room; PD, Parkinson's disease.

We found 12 studies on hospital admissions (Table 3): 11 studies totalling 3216 PD/parkinsonism patients,^4^,^9^,^10^,^13^,^15^–^19^,^21^,^22^ and one study^20^ on a database with 15304 PD/parkinsonism patients. In these studies, PD patients are hospitalized in frequencies ranging from 7–28% per year.^9^,^10^,^15^,^19^,^20^

**TABLE 3:** Hospital admissions

Study	Inclusion	Exclusion	Number	Design	Control group*	Hospital admissions	Five most frequent admission reasons
Vossius et al., 2010^15^	PD		108 patients	Prospective	Yes	All selected patients	PD related symptoms 25%
							Vascular disorders 14%: significant less than control group
							Pulmonary disorders including pneumonia 12%
							Trauma 12%: significant more than control group
							Cancer 7%: significant less than control group
							Remark: discharge diagnosis instead of admission diagnosis
Klein et al., 2009^16^	PD		143 patients	Retrospective	No	All selected patients	Motor complications 37%
	Emergency admissions to Neurological Department						Psychosis 24%
							Somatic problems 14%
Guneysel et al., 2008^13^	PD	Admissions resulting in death	76 patients	Prospective	No	All selected patients	Trauma 28%
	Emergency hospital admission	PD diagnosis during ER visit					UTI 20%
							Cardiovascular 15%
							Pneumonia, cerebrovascular both 12%
							GI 8%
Vargas et al., 2007^9^	PD	Hoehn andYahr stage 5	144 patients	Retrospective	No	28% programmed admissions in 1 year	Complication treatment
		Parkinsonism					Drug adjustment
		Severe cognitive dysfunction					
Louis et al., 2007^17^	Young-onset PD	Obstetrical admissions	714 patients	Retrospective	Yes	All selected patients	Psychosis 23%: significant more than control group
	18-40 years old						Craniotomy 7%: significant more than control group
							Pneumonia, UTI both 6%
							Headache or seizure 4%
							Rehabilitation 3%: significant more than control group Remark: discharge diagnosis instead of admission diagnosis
Temlett and Thompson, 2006^18^	PD		761 hospital admissions	Retrospective	No	All selected patients	Primary for PD 15%
							Falls and fractures 11%
							Pneumonia, cardiac disease both 10%
							GI 9%
							Organic brain syndrome 6%
Cosentino et al., 2005^10^	PD	Parkinsonism	130 patients	Retrospective	No	19% in 1 year	Diseases of digestive system 17%
		Admissions related to PD					Diseases of circulatory system, rehabilitation both 14%
							Cataract 10%
							Injury, chest pain both 7%
							Sleep disturbance, epistaxis, abdominal pain, osteoarthrosis: all 3%
Woodford and Walker, 2005^19^	PD	Parkinsonism	367 patients	Retrospective	No	35% in 4 year	Cardiovascular 20%
	Emergency hospital admission	Elective admissions					Falls 13%
		Day-case procedures					Pneumonia 11%
		Admissions resulting in death of patient					UTI 9%
							Decreased mobility/ dyskinesia, psychiatric both 8%
Guttman et al., 2004^20^	PD	< 25 years of age	15304 patients	Retrospective	Yes	68% in 6 years	Compared with controls
	Use of PD drugs						First: aspiration pneumonia
							Second: affective psychosis
							Third: hip fractures
							Fourth: urinary tract disorders including infections
							Fifth: septicemia
Martignoni et al., 2004^4^	PD	Parkinsonism	132 patients	Prospective	No	All selected patients	Admission to neurological department
							Poor control of PD symptoms 37%
							Neurological 25%
							Diagnosis confirmation, psychiatric complaints both 13%
							Sudden worsening of motor symptoms 7%
							Head trauma and fracture 2%
							Admission to non-neurological department
							Medical and infectious illnesses 27%
							Traumas with fracture 24%
							Cardio-circulatory 22%
Tan et al., 1998^21^	PD	Drug induced parkinsonism	173 patients	Retrospective	No	All selected patients	Chest infection 22% Falls 13%
							Control of PD symptoms 10%
		Parkinson-plus syndromes					General medical problems 9%
Urinary dysfunction 8%							
Kessler 1972^22^	PD		468 patients	Retrospective	Yes	All selected patients	PD 19%
	Paralysis agitans						Circulatory system 16%
	Parkinsonism						Digestive system 10%
							Accidents 9%
							Respiratory system 8%
							Remark: Neoplasm 4%: significant less than control group

* Comparison with inclusion group concerning admission reasons.

PD, Parkinson's disease, UTI, urinary tract infection, GI, gastrointestinal.

PD/parkinsonism patients are hospitalized approximately one and a half times more frequently^20^,^23^,^24^ and generally 2 to 14 days longer^12^,^16^,^17^,^19^,^21^,^24^–^29^ than non-PD patients, although there is not a difference in every study.^15^,^30^

Reasons for emergency and hospital admission can be divided in:

1. Direct disease related morbidity: motor complications, psychiatric symptoms, autonomic dysfunction, sensory symptoms, sleep disorders, and side effects of anti-parkinsonian drugs.
2. Indirect disease related morbidity: traumas and pneumonia.
3. Non-PD related causes.

As most studies vary greatly in selection of patients, exact relative proportions of these 3 groups cannot be assessed (Tables 2 and 3). Some studies found that PD patients are more likely to be admitted to the ER and hospital for complications of the disease and its management than for primary motor problems.^13^,^18^ A part (5–21%) of the patients were first diagnosed to have PD during a hospitalization.^19^,^21^

### Problems During Hospitalization

We found one prospective study on acute events occurring during hospitalization.^4^ When admitted to a neurology ward (83 PD patients, mean age 69 years, mean disease duration 6 years), patients received an average of 0.6 non-neurological consultations. Reasons were: (aspiration) pneumonia, urinary infections and retention, diarrhoea, atrial fibrillation, postural hypotension, low back pain, and TIA. Specialists consulted most frequently were cardiologists, internal medicine specialists and orthopaedic surgeons. Acute events observed during hospitalization on non-neurological department of 20 patients (mean age 80 years) after ER visits were: Urinary infection (33%), agitation, confusion and hyperthermia (28%), pressure ulcers and leg oedema (22%), bowel occlusion and hypotension (11%), and dysphagia (6%). Mortality rate during hospitalization of this last group was 20%. We found one retrospective study on this subject (173 PD patients), but this study is unclear on the presence or absence of complications before the hospital admission.^21^ Problems identified in this study do accord however with those described above.^4^

Another retrospective study shows that in 74% of 35 emergency hospital admissions of PD patients, their medication was stopped, omitted or prescribed inappropriately with 61% of this group suffering significant sequelae. Nonadherence to medication schedules was a large problem and in 11% of cases antidopaminergic medication (metaclopramide) was prescribed.^31^

In a study analyzing inpatient falls, the use of anti-Parkinson's medication was an evident risk factor (OR 5.04).^32^

Apart from the studies above, we found several opinionated views and reviews on problems during hospitalization of PD patients. These authors point to disease fluctuations, stress,^7^ and medications like butyrophenones, phenothiazines, and metoclopramide, prescribed during hospitalization, as possible causes of PD exacerbation.^6^,^33^–^38^ Additionally, sleep disorders, verbal communication problems, nutritional intake difficulties, and cognitive changes are mentioned as problem areas.^7^,^19^,^39^

### Perioperative Problems

We found 15 retrospective studies on problems during and after surgery in patients with PD, with an emphasis on the postoperative period. Of these 15, two retrospective studies included a control group of non-PD patients.^25^,^27^ One retrospective study focused on postoperative confusion^33^ and two retrospective studies on medication errors.^40^,^41^ Another study with 10 patients is actually a case report with 10 PD patients,^42^ and the remaining 9 papers are retrospective studies on PD patients undergoing orthopaedic surgery.^29^,^43^–^50^

The first study compared 234 PD patients with a control group of 40,979, undergoing elective bowel resection, cholecystectomy, or radical prostatectomy, during a 6-year period. PD patients had a significantly increased incidence of aspiration pneumonia, urinary-tract infection, bacterial infections, with odds ratios of 3.8, 2.0, 1.7, respectively. Odds ratios for postoperative delirium and hypotension in PD patients were 2.6 and 2.5, with lesser significance. PD patients had a mortality rate of 7.3%, compared with a 3.8% in the control group.^25^

In the second study with a non-PD control group, 51 PD patients, treated on different surgical departments, were compared using matched-pair analysis. There were significantly more postoperative falls in the PD group (18% vs. 4%), and a higher although not significantly increased number of urinary tract infections (33% vs. 24%), pneumonia (10% vs. 4%), and also wound infection, urinary retention, respiratory insufficiency, and postoperative confusion. PD patients were hospitalized for more days and stayed on the intensive care unit longer.^27^

Another retrospective study showed that 60% of 25 PD patients with no pre-existent mental status abnormalities suffered postoperative confusion, some with hallucinations. The onset of the delirium was often delayed, 70% after 36 hours. In this study, there was no relationship between delirium and type of anti-Parkinson medication or anaesthetic procedure.^33^

In a retrospective study on pharmacological management during 51 surgical admissions of PD patients or patients with parkinsonian syndromes treated with PD medication, 71% had missed doses of their medication. Overall, antidopaminergic medication was prescribed in 41% and administrated in 22% although not allowed. 47% (69% for non-day-cases admissions) had complications: neuropsyciatric 41%, falls 8%, and worsening of motor symptoms 5%.^40^ A second study on pharmacological management found that 30% of 92 PD patients had medication administration problems, leading to an increase in postoperative confusion or worsening of PD: 84% to 36% in the well-managed group. Overall 31% of patients was dissatisfied in the way their PD was managed in the perioperative period.^41^

In the smallest study (n = 10), all patients had complications. This paper identified the same problems as mentioned above, and additionally found pressure sores as an important problem.^42^

Next to the above studies, there are nine retrospective research articles on orthopaedic surgery and its complications. These studies (totalling 433 PD patients, Table 4) found pneumonia, urinary tract infections, confusion, and decubitus as the most frequent postoperative complications, in frequencies up to 49%^44^–^50^ with an overall six month mortality up to 47%.^44^,^45^,^47^,^48^,^50^,^51^ Apart from complications, several studies are contradictory regarding the outcome of orthopaedic procedures in PD patients,^26^,^29^,^30^,^43^–^60^ and postoperative rehabilitation is reported to be slower.^28^,^55^

**TABLE 4:** Orthopedic surgery, complications

Study	Inclusion	Exclusion	Number	Design	Intervention	Most frequent complications
Mehta et al., 2008^29^	PD	Total knee arthroplasty revision	34 patients	Retrospective	Total knee arthroplasty	Confusion 35%
			39 knees			Superficial wound infection, aspiration pneumonia both 6%
Weber et al., 2002^48^	PD		98 patients	Retrospective	Hip replacement	6 Month mortality 6%
			107 hips			Overall complications: 36%
						UTI 7%
						Dislocation 6%
						Postoperative confusion 4%
						Pneumonia, deep venous thrombosis both 3%
Duffy and Trousdale, 1996^43^	PD		24 patients	Retrospective	Total knee arthroplasty	Confusion 20%
			33 knees			Deep venous thrombosis, superficial infections both 8%
						Myositis ossification, urinary retention, wound necrosis, respiratory tract infection all 4%
Turcotte et al., 1990^50^	PD		87 patients	Retrospective	Hip fracture surgery	After 6 months:
			94 hips			Mortality 14%: myocardial infarction (n=5), infection (n=2), pulmonary embolism (n=1), unknown (n=4)
						Orthopaedic problem 14%
						Decubitus ulcers 5%
						Wound infections 4%
Vince et al., 1989^46^	PD		9 patients	Retrospective	Total knee arthroplasty	Deep vein thrombus (n=4), UTIs (n=3), temporary disorientation (n=2), skin necrosis (n=1), intestinal ileus (n=1), pulmonary embolism (n=2)
			13 knees			
Staeheli et al., 1988^45^	PD	Parkinsonism	49 patients	Retrospective	Hip fracture surgery	6 Months complication:
			50 hips			Mortality 20%: pneumonia 40%, congestive heart failure 20%, cerebrovascular accident 20%, pulmonary embolism 10%, breast cancer 10%
						UTI 20%, pneumonia 10%, decubitus ulcers 10%, pulmonary embolism 6%, cerebrovascular accident 6%, wound infection 4%
Eventov et al., 1983^44^	PD	Impacted subcapital fractures	62 patients: 45 patients undergoing surgery	Retrospective	Hip fracture surgery	3 Month mortality surgery group 31% (1 year 38%): bronchopneumonia 43%, congestive heart failure 21%
	Ambulatory					3 Month mortality in patients not undergoing surgery 29% (35% 1 year)
						Survivors surgery group (n=31):
						UTI 23%, decubitus 23%, bronchopneumonia 16%, contractures 6%, deep infection, cardiac arrythmias, myocardial infarction, dislocation, thrombophlebitis, paralytic ileus all 3%
						Survivors of patients not undergoing surgery (n=12):
						UTI 17%, decubitus 25%, bronchopneumonia 0%, contractures 17%
Coughlin and Templeton, 1980^47^	PD		47 patients	Retrospective	Hip fracture surgery	6 Month mortality 47%
			49 hips			Decubitus 49%
	Ambulatory					Dislocation 37% (endoprosthesis)
Rothermel and Garcia, 1972^49^	PD		23 patients: 16 without levodopa, 7 with levodopa	Retrospective	Hip fracture surgery	With levodopa: phlebitis n=1
						Without levodopa: debrided decubitus ulcers n=2, phlebitis n=2, deep hematoma n=2, dislocation n=2, urinary septicaemia n=1, fatal myocardial infarction n=1

PD, Parkinson's disease; UTI, urinary tract infection.

Next to these more or less formal surveys, case report and reviews mention the following perioperative problems: neuroleptic malignant syndrome, medication- or anaesthetic-induced exacerbation of PD, side-effects of PD medication, postextubation laryngeal spasm, bronchospasm, respiratory arrest, difficulty with salivation, gastrointestinal complications, deep vein thrombosis, urinary disturbances, temperature regulation problems, and tremor hampering eye surgery.^6^,^8^,^34^–^38^,^61^–^80^

### Improvement of Care for the Hospitalized PD Patient

#### Improvement During Hospitalization

There are no studies analyzing the effects of suggested recommendations/improvements. Some authors favour a multidisciplinary approach during hospitalization.^4^,^7^ Other suggestions are: continuing the exact personal medication regime, education of nurses and doctors, being attentive for early signs of complications like pneumonia to start early treatment, falling prevention, (temporary) medication adjustment, emotional support, good sleep hygiene by maintaining the home bedtime and trying to prevent sleeping during the day, sometimes consulting a sleep disorder specialist or start sleeping medication, exercise, speech therapy, sufficient nutritional intake high in fibre, adequate fluid intake, preventing a confusional state by limiting the number of care-givers and the amount of light and noise during night sleeping, avoiding certain medication harmful for the PD patient, and consulting a neurologist.^7^,^21^,^31^,^35^,^37^,^67^

#### Improvement of Perioperative Care

There are only two studies on medication continuation during surgery^81^,^82^ and one study analyzing the effect of an early postoperative neurologic consultation,^29^ and numerous case reports, and opinionated views and reviews.

During preoperative screening, some authors recommend extra attention to be paid to this group of patients, especially to respiratory status, urologic system, fluid status, cardiovascular system, gastrointestinal system, autonomic system, and cognition.^6^,^25^,^35^,^37^,^66^,^73^,^74^,^78^,^83^ And, if necessary, supplemented with additional diagnostics like laboratory tests, pulmonary tests, electrocardiogram, and X-ray.^25^,^66^,^73^,^78^ Most of the literature describes measures with regard to PD medication in the intraoperative period. To prevent large descents of dopamine levels intra- and postoperatively many authors advise continuation of PD medication as long as possible preoperatively and resume it as soon as possible postoperatively, and PD medication is preferably continued.^6^,^8^,^25^,^34^,^37^,^38^,^42^,^67^–^70^,^72^–^74^,^76^,^78^,^79^,^81^,^82^,^84^–^92^ Two studies on this topic describe PD patients using the rotigotine transdermal patch, a nonergot D1/D2/D3 dopamine agonist.^81^,^82^ In the first prospective study, oral dopaminergic medication was easily switched to rotigotine before surgery and resumed afterwards in 14 PD patients undergoing surgery under general anaesthesia. Adverse events were two dopaminergic side effects namely nausea and hallucinations and one ventricular asystole.^81^ The second study on this topic describes PD patients derived from two prospective clinical trails. PD patients undergoing surgery under general anaesthesia and who continued using rotigotine during surgery were retrospectively analyzed (n = 25). There was no worsening of PD symptoms, but (only) three complications: deep vein thrombosis, infection, and pain.^82^

As other substitutes for dopaminergic medication intraoperatively, continuous intravenous levodopa infusion, continuous subcutaneous infusion or immediate postoperative injections of apomorphine, and enteral levodopa/carbidopa via nasogastric tube or duodenostomy have been used by various authors,^68^,^69^,^72^,^76^,^78^,^88^,^92^,^93^ but none have been studied in a controlled trial. The use of parenteral anticholinergic and antihistaminic medication as anti-Parkinson therapy is limited according to some authors, and may aggravate postoperative discomfort because of autonomic side effects and confusion.^72^,^76^,^78^

Some authors claim that general anaesthesia should be avoided when possible, and prefer local anaesthesia.^37^,^66^,^67^,^73^,^75^,^90^ This is supported by a case report of a PD patient, undergoing surgery with regional anaesthesia, who was successfully given levodopa and carbidopa orally during the operation.^86^ Regional anaesthesia also avoids postoperative nausea and vomiting.^73^ Less invasive interventions, like laparoscopic surgery over abdominal surgery, are recommended.^66^ Some authors advocate carefully considering the used operation technique.^47^,^50^

In a retrospective study, the postoperative period was analyzed in PD patients undergoing total knee arthroplasty receiving a preoperative or immediate postoperative neurologic consultation (n = 13) compared with patients receiving a delayed or no visit (n = 21). Only in the first group, there was a significant improvement in total Unified Parkinson's Disease Rating Scale with most improvement in activities of daily living but also an improvement of mood, mentation and behavior, and motor examination. In this group, there was also a shorter length of stay (*P* < 0.01) and less patients were confused: 15 to 48%.^29^

There are many more recommendations, without formal studies. Postoperatively lung expansion manoeuvres, noninvasive mechanical ventilation, percussion, adequate pain control, aspiration precautions, (early) mobilization, recognition of unique physical limitations and medication combinations, physiotherapy, turning regimens, maintenance of volume status, antiparkinsonian therapy adjustments, analyzing urine for urinary tract infections, and deep venous thrombosis prophylaxis may prevent complications like pulmonary emboli, infections, deep vein thrombosis and decubitus.^6^,^27^,^42^–^45^,^50^,^55^,^59^,^60^,^66^,^72^,^74^,^78^,^83^,^91^,^94^–^96^ In case of prolonged endotracheal intubation early tracheostomy is preferred.^66^ Except for two case reports, there is no literature concerning the pre-emptive administration of drugs to prevent complications. In one case report, the cholinesterase inhibitor rivastigmine was given preoperatively to prevent delirium postoperatively in a PD patient successfully treated with this drug in two previous delirious episodes.^97^ In another case report, prokinetics were administered in two patients to prevent paralytic ileus.^92^ Some authors suggest preventive antibiotics to prevent infections.^44^,^45^,^59^ As described before, the onset of the postoperative delirium is often delayed. If patients are discharged rapidly after surgery, there should be sufficient support in the home environment.^33^ Overall, team work between different specializations, like surgery, neurology, geriatrics, and a rehabilitation unit is generally advocated.^28^,^96^,^98^

## Discussion

There are few studies analyzing the problems a PD patient encounters during hospitalization, and there are even less studies analyzing possible solutions. Most studies are retrospective and have small numbers of patients. In some studies, PD patients were diagnosed according to clear diagnostic criteria (like the United Kingdom Parkinson's Disease Society Brain Bank's clinical diagnostic criteria),^4^,^9^–^11^,^15^,^19^,^48^ but in most studies, the diagnostic criteria were not clearly mentioned: a neurologist confirmed the diagnosis,^13^,^43^,^45^,^46^,^48^ or medical record systems and/or patients notes were used to identify PD patients,^17^,^20^–^22^,^25^,^27^,^33^,^40^ or no information according to the diagnostic criteria, except the use of PD medication, was given.^16^,^31^,^41^,^42^,^47^,^49^,^50^,^81^,^82^

Overall PD patients are more frequently^20^,^23^,^24^ and longer^12^,^16^,^17^,^19^,^21^,^24^–^29^ hospitalized compared with controls. Generally the leading causes for admission are injuries (many with fractures), infections [mainly pneumonia and urinary tract infections (UTI)], poor control of PD and complications of PD treatment, psychiatric disturbances, and diseases of the circulatory and digestive system. A reduction of the number of admissions might be achieved by extra attention to fall prevention, adequate drug regulation with acuity for side effects, preventing and recognizing of early symptoms of infections and active monitoring in the home situation of both patients' vital parameters and therapy compliance.^5^,^18^,^20^ When admitted to a hospital, most PD patients stay on a general medicine or surgery ward instead of a neurological one.^5^,^15^ Apart from one small prospective study^4^ and a retrospective study,^31^ little is known about problems occurring during hospitalization of PD patients not undergoing surgery, mentioning the usual direct and indirect PD related problems and medication issues.

There are more studies on complications in PD patients hospitalized for surgery, particularly in the postoperative period. (Aspiration) pneumonia, UTI, bacterial infections, postoperative falls, postoperative delirium/confusion (often with a delayed onset^33^), and hypotension occur more frequently in this group of patients than in controls.^25^,^27^ Pressure sores are also an important complication.^42^ Mortality rates are also higher.^25^ These data are confirmed by studies on PD patients undergoing orthopaedic surgery.^29^,^43^–^50^ Many PD patients had postoperative medication administration problems with more postoperative confusion or worsening of PD as a result.^40^,^41^ It is striking that almost one third of the PD patients were dissatisfied concerning their PD treatment.^41^

### Suggestions for Improvement of Hospital Care

There are many proposals for improvement during hospitalization in PD patients with or without surgery. Suggestions on improvement do vary, but most authors agree that attention should be given to all aspects of PD and not only to motor function.

Most publications refer to the intraoperative period mainly to prevent a decline in dopamine levels because of discontinuation of dopaminergic medication, including two studies favouring the use of a rotigotine patch.^81^,^82^ Most authors agree that anesthesiologists and surgeons should take the increased vulnerability of PD patients into account when planning and selecting procedures.^37^,^47^,^50^,^66^,^67^,^73^,^75^,^86^,^90^ One small study shows that early consultation by a neurologist may prevent complications and reduce length of hospital stay.^29^

## Conclusions

Most studies were retrospective and had small numbers. Prospective studies with large numbers of PD patients, defined according to clear diagnostic criteria and preferable diagnosed by a specialist with special interest in PD, would be preferred for future research. Generally patients with PD are hospitalized much more frequently and longer than control groups. The leading causes are injuries, infections, poor control of PD and complications of PD treatment. The inclusion of hospitalization data into patient registries for PD could be a major improvement in identifying the important problems.

There are many (un-researched) proposals for improvement during hospitalization in PD patients. A substantial number of PD patients' admissions might be prevented. There should be guidelines concerning the hospitalized PD patients, with accent on early neurological consultation or consultation of another specialist like a geriatrician with a special interest in PD and team work between different specialities, and on sufficient training of all people involved in the treatment and recovery of this patient group. This protocol should include dopaminergic replacement therapy in case PD patients are not allowed or able to take their oral medication. Preferably this therapy should not be laborious and not invasive so that it is easily applicable.
