# Supplementary material for: Clinical Problems in the Hospitalized Parkinson's Disease Patient: Systematic Review
Source: Mov Disord. 2011 Jan 31;26(2):197–208. doi: 10.1002/mds.23449 (PMC3130138; doi:10.1002/mds.23449)
Supplement: Supplementary file 1 [file mds0026-0197-SD1.doc]

**Author Roles**

OHHG and WEJW participated in design and data collection, interpretation of the data, and prepared the manuscript. AW participated in manuscript preparation and revisions and helped to bring the manuscript to its final version. All authors have read and approved the final manuscript.

Full Financial Disclosures of all Authors for the Past Year

None for all authors
